# Does experience change the role of systematic biopsy during MRI-fusion biopsy of the prostate?

**DOI:** 10.1007/s00345-023-04564-z

**Published:** 2023-08-25

**Authors:** Matthias Jahnen, Thomas Amiel, Tobias Wagner, Florian Kirchhoff, Jakob W. Büchler, Charlotte Düwel, Florestan Koll, Kay Westenfelder, Thomas Horn, Kathleen Herkommer, Valentin H. Meissner, Jürgen E. Gschwend, Lukas Lunger

**Affiliations:** 1 Department of Urology, University Hospital Rechts Der Isar, Technical University of Munich, Ismaninger Str. 22, 81675 Munich, Germany; 2 Department of Urology, University Hospital Frankfurt, Theodor-Stern-Kai 7, 60590 Frankfurt Am Main, Germany; 3 Department of Urology, Spital STS AG, Krankenhausstrasse 12, 3600 Thun, Thun Switzerland

**Keywords:** Prostate cancer, MRI-targeted prostate biopsy, Systematic biopsy, Residents in urology

## Abstract

**Purpose:**

To determine the role of biopsy experience regarding a potential benefit of additional systematic biopsies and fusion failures during MRI-targeted biopsy of the prostate.

**Subjects/patients and methods:**

We retrospectively evaluated 576 men undergoing transrectal (MRI)-targeted biopsy of the prostate by seven residents in urology between November 2019 and March 2022. Benefit of systematic biopsies (detection of ISUP ≥ 2 PCa (clinically significant PCa (csPCa)) solely in systematic biopsies) and fusion failure (detection of csPCa during systematic biopsies in the area of a reported MRI-lesion and no detection of csPCa in targeted biopsy) were compared by growing biopsy experience levels. Multivariable regression analyses were calculated to investigate the association with benefit of systematic biopsies and fusion failure.

**Results:**

The overall PCa detection rate was 72% (413/576). A benefit of systematic biopsies was observed in 11% (63/576); of those, fusion failure was seen in 76% (48/63). Benefit of systematic biopsies and fusion failure were more common among residents with very low experience compared to highly experienced residents (18% versus 4%, *p* = 0.026; 13% versus 3%, *p* = 0.015, respectively). Increasing biopsy experience was associated with less benefit from systematic biopsies (OR: 0.98, 95% CI 0.97–0.99) and less fusion failure (OR: 0.98, 95% CI 0.97–0.99).

**Conclusions:**

The benefit of systematic biopsies following targeted biopsy decreases with growing biopsy experience. The higher risk of fusion failure among inexperienced residents necessitates systematic biopsies to ensure the detection of csPCa. Further prospective trials are warranted before a targeted only approach can be recommended in routine clinical practice.

## Introduction

To date, multi-parametric magnetic resonance imaging (mpMRI) of the prostate is recommended for men with suspected prostate cancer (PCa) before undergoing prostate biopsy (bx) [13, 21, 23]. mpMRI has substantially facilitated clinical decision-making regarding prostate bx and reduced the detection of clinically non-significant PCa (defined as ≤ International Society of Urological Pathology classification (ISUP) 1 PCa) [2, 5, 13]. Although the use of magnetic resonance imaging (MRI)-targeted bx has been shown to be non-inferior to systematic bx in patients with elevated PSA, there is no final consensus as to whether systematic bx may be safely omitted [6, 7, 13, 20]. Given that 5–10% of clinically significant PCa (csPCa) (defined as ≥ ISUP 2 PCa) could be missed by MRI-targeted bx alone, an additional 10–12 core systematic bx is recommended in all bx naïve patients and routinely carried out at our clinic [21]. During the combination of systematic and MRI-targeted bx commonly, more than 12 and up to 25 bx cores are extracted, potentially increasing the risk of complications, discomfort, and peri-prostatic fibrosis [9, 15, 22, 24]. Therefore, strategies to reduce the number of bx cores have been increasingly investigated and are warranted [1, 4, 11, 14].

Available data indicate that systematic bx could be omitted in patients with a Prostate Imaging Reporting and Data System (PI-RADS) score of 5 on mpMRI and previous negative bx [1, 4]. However, successfully performing MRI-targeted bx of the prostate may not only depend on patient and mpMRI characteristics but may also substantially depend on the experience of the performing urologist. We hypothesized that omitting systematic bx may not be safe for every urologist, especially when the rate of procedural experience is low. Recent studies underscored that a higher level of experience is associated with reduced procedure durations, a higher rate of csPCa detected, and an improved accuracy of MRI-targeted bx [10, 12, 16, 25, 26, 30]. This aspect appears particularly important given that bx of the prostate may be performed by residents in urology rather than only by seasoned specialists. Still, most studies assessing bx experience are limited by either a low number of included bx (< 300 bx) [12, 16, 25, 26] or a low number of included urologists performing the bx (< 5 urologists) [12, 16, 19, 25, 29].

Therefore, this real-world study aimed to investigate the role of growing procedural experience regarding a 1) potential benefit of the additional systematic bx as well as regarding 2) potential fusion failures during MRI-targeted bx in a large sample of 576 MRI-targeted bx performed by 7 residents in urology.

## Subjects/patients and methods

### Design and procedure

The present retrospective analysis included *n* = 576 patients undergoing transrectal MRI-targeted and systematic bx of the prostate for suspected PCa (elevated PSA > 4 ng/ml and/or abnormal digital rectal examination and at least PIRADS ≥ 2 lesion on mpMRI) between November of 2019 and March of 2022 at the Department of Urology of the Klinikum rechts der Isar, Technical University of Munich. The study was approved by the Ethics Committee of the Technical University of Munich in February 2022 (Trial number 2023-74-S-KH) and followed the Declaration of Helsinki Ethical Principles for Medical Research.

### mpMRI and biopsy strategy

All mpMRI scans were evaluated according to the PI-RADS v2.0 protocol [27]. Detection of csPCa was defined as PCa > ISUP 1 upon histopathological analysis. All men received at least two MRI-targeted bx and 12-core systematic bx (6 cores extracted from each lobe from the lateral and medial portion of the base, mid portion and apex of the prostate). The digital fusion of ultrasound and mpMRI was performed using the Canon Aplio i800 ultrasound system. All bx were performed by residents without prior experience in performing prostate bx prior to the assessed time period.

### Sociodemographic and clinical characteristics

The following sociodemographic and clinical data were obtained for this analysis: age at bx, PSA level at bx, prostate volume, prior bx (yes (negative); yes (positive/active surveillance); no), max. PI-RADS score (≤ 3, 4, 5), number of mpMRI targets, number of total cores, number of MRI-targeted bx cores, number of positive cores, grading.

Bx experience was defined individually for each bx as the total number of procedures performed by the resident up until the current procedure.

### Benefit of systematic bx, benefit of targeted bx, and fusion failure

Benefit of systematic bx was defined as the detection of an ISUP ≥ 2 PCa in the systematic bx and either (a) no detection of PCa or (b) detection of ISUP 1 PCa in the MRI-targeted bx.

Benefit of targeted bx was defined as detection of an ISUP ≥ 2 PCa in the targeted bx and either (a) no detection of PCa or (b) detection of ISUP 1 PCa in the systematic bx.

Fusion failure was defined as the detection of csPCa (ISUP > 1) during systematic bx in the area of a reported mpMRI lesion and no detection of cancer in the MRI-targeted bx supposedly targeting this mpMRI lesion. All prostate regions unilaterally adjacent to the region containing the suspected lesion on mpMRI were considered “areas of the reported mpMRI lesion” (e.g. in cases of a mpMRI lesion located in right lateral prostate apex, systematic biopsies extracted from the right apex and the right lateral mid portion of the prostate were considered in the area of the reported mpMRI lesion).

### Statistical analysis

Descriptive statistics were calculated for all study variables (Mean and standard deviation (SD) or median and interquartile range (IQR) or range). Bx experience levels were clustered into quintiles using visual binning (very low experience: 1st–14th (*n* = 123), low experience: 15th–28th (*n* = 112), moderate experience: 29th–47th (*n* = 116), high experience: 48th–71th (*n* = 112), and very high experience > 71th (*n* = 113) bx). Chi-Square tests were calculated to determine differences between binned bx experience categories regarding benefit of systematic bx, benefit of targeted bx, fusion failure, and overall PCa detection rate.

Univariable and multivariable logistic regression analyses were calculated to identify variables associated with (1) benefit of systematic bx following MRI-targeted bx and (2) fusion failure. Statistical significance was set at *P* < 0.05. All analyses were performed using SPSS (Version 26, IBM, Armonk USA). Graphs were created using Microsoft Office (Version 16.66.1, Microsoft, Redmond USA).

## Results

### Patient characteristics, mpMRI, and bx results

A total of 576 men (age (SD): 66 (10) years) were evaluated for this retrospective study. Of those, the majority (52% (298/576) had a PI-RADS 4 lesion and 23% (132/576) had a PI-RADS 5 lesion on mpMRI. The overall PCa detection rate was 72% (413/576) (csPCa 90% (373/413)). PCa was found in 92.4% (122/132; csPCa: 91.6%) of men with a PI-RADS 5 lesion, in 79.5% (237/298; csPCa: 71.8%) of men with PI-RADS 4 lesion and 35.7% (40/112; csPCa: 23.2%) in men with a PI-RADS 3 Lesion. Detailed patient characteristics are shown in Table 1.

**Table 1:** Sociodemographic and clinical characteristics of the study sample (*N* = 576)

Characteristic	
Mean (SD) age at biopsy, *years* (*n* = 576; missing: 0)	66 (10)
Median PSA (*ng/ml)* (IQR) (*n* = 576; missing: 0)	7 (5–10)
No. max. PI-RADS Score (%) (*n* = 576; missing: 0)	
≤ 3	146 (25)
4	298 (52)
5	132 (23)
No. DRE (%) (*n* = 505; missing: 71)	
Suspicious	147 (29)
Not suspicious	358 (71)
Median prostate volume, *ml* (IQR) (*n* = 506; missing: 70)	45 (35–60)
No. prior biopsy (%) (*n* = 576; missing: 0)	
Yes (negative)	73 (13)
Yes (positive/active surveillance)	9 (2)
No	494 (85)
Median number of MRI lesions (Min–Max) (*n* = 576; missing: 0)	1 (1–4)
Mean (SD) number of total cores extracted (*n* = 576; missing: 0)	15 (2)
Mean (SD) number of fusion cores extracted (*n* = 576; missing: 0)	4 (2)
No. patients with prostate cancer (%) (*n* = 576; missing: 0)	
Yes	413 (72)
No	163 (28)
No. tumor grading (%) (*n* = 413; missing: 0)	
ISUP 1	40 (7)
ISUP 2	185 (32)
ISUP 3	100 (17)
ISUP 4	64 (11)
ISUP 5	24 (4)

*DRE* digital rectal examination, *IQR* inter quartile range, *ISUP* International Society of Urological Pathology, *Min–Max* minimum–maximum, *No.* number, *PSA* prostate specific antigen, *SD* standard deviation

### Benefit of systematic or MRI-targeted bx

A benefit of systematic bx was observed in 11% (63/576) of all cases. In other words, of the 373 men diagnosed with csPCa (> ISUP 1), 63 (17%) men had a csPCa detected only by systematic bx. These were either ISUP 2 (95%, (55/63)) or ISUP 3 (5%, (8/63)) PCa. A potential fusion failure was present in 76% (48/63) of these bx. A benefit of targeted bx was observed in a total of 5% (29/576) of all bx, meaning that 8% (29/373) of all csPCa was solely detected by targeted bx.

### The role of experience during systematic and MRI-targeted bx of the prostate

A total of 7 residents in urology performed the bx analyzed in this study. Each resident performed at least 28 bx. The median bx experience (IQR) at the end of the observed time frame was 66 (38–75).

**Fig. 1:**
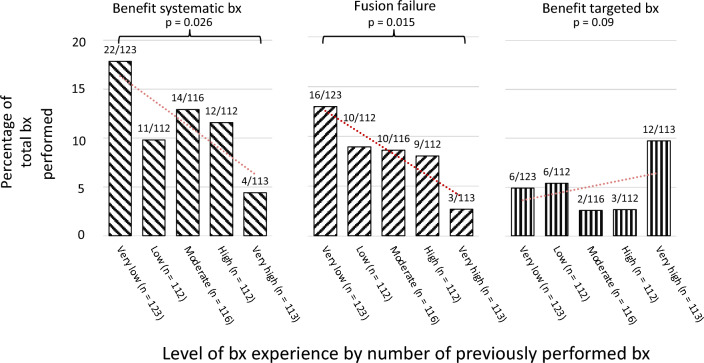
Benefit of systematic biopsy, fusion failure, and benefit of targeted biopsy across growing biopsy experience (red dotted line represents the trend line). *Bx* biopsy

A benefit of systematic bx was observed significantly more often among the least experienced residents as compared to residents with the highest experience (18% versus 4%, respectively, *P* = 0.026, Fig. 1). Fusion failures were more frequent among the least experienced residents compared to very experienced residents (13% versus 3%, respectively, *P* = 0.015, Fig. 1).

No statistically significant differences in the detection rate of csPCa among men with PI-RADS 4 or 5 lesions stratified by experience levels were found (very low experience: 87% versus low experience: 78% versus moderate experience: 87% versus high experience: 83% versus very high experience: 83%, *P* = 0.4).

Multivariable regression analysis revealed that growing bx experience was independently associated with lower benefit from systematic bx of the prostate (OR 0.98, 95% CI 0.97–0.99) and lower risk of fusion failure (OR 0.98, 95% CI 0.97–0.99). Higher PSA was associated with lower risk of fusion failure (OR 0.89, 95% CI 0.81–0.99), whereas a higher number of extracted biopsy cores was associated with higher risk of fusion failure (OR 1.23 95% CI 1.01–1.50) (Table 2).

**Table 2:** Univariable and multivariable regression to determine the association of selected clinical parameters with benefit from additional systematic biopsies during MRI-targeted biopsy and fusion failure

Clinical parameters	Benefit systematic biopsy				Fusion failure			
	Univariable regression		Multivariable regression		Univariable regression		Multivariable regression	
	OR (95%-CI)	*P*-value	OR (95%-CI)	*P*-value	OR (95%-CI)	*P*-value	OR (95%-CI)	*P*-value
Age (continuous^1^)	0.98 (0.95–1.00)	0.11	**–**	**–**	0.98 (0.95–1.01)	0.18	**–**	**–**
PSA (continuous^1^)	**0.94 (0.88–1.00)**	**0.049**	0.95 (0.89–1.00)	0.06	**0.91 (0.83–0.99)**	**0.045**	**0.89 (0.81–0.99)**	**0.030**
PI-RADS-Score (ref. 5)								
2	1.60 (0.40–6.42)	0.82	1.15 (0.28–4.71)	0.84	0.58 (0.07–4.94)	0.62		-
3	1.01 (0.36–2.90)	0.69	0.70 (0.24–2.05)	0.51	0.69 (0.19–2.43)	0.56		-
4	**2.98 (1.20–6.50)**	**0.006**	2.16 (0.96–4.85)	0.06	1.64 (0.68–3.97)	0.27		-
DRE (ref. suspicious)								
Not suspicious	0.54 (0.26–1.10)	0.09	–	–	0.83 (0.38–1.83)	0.64	–	-
Prostate volume (continuous^1^)	0.99 (0.99–1.01)	0.81	–	–	0.99 (0.98–1.01)	0.76	–	-
Prior biopsy (ref. no)								
Yes (negative)	0.80 (0.35–1.82)	0.59	–	–	0.34 (0.08–1.36)	0.13	–	-
Biopsy cores taken (continuous^1^)	1.08 (0.92–1.26)	0.35	–	–	**1.23 (1.01–1.49)**	**0.042**	**1.23 (1.01–1.50)**	**0.043**
Experience (continuous^1^)	**0.98 (0.97–0.99)**	**0.005**	**0.98 (0.97–0.99)**	**0.003**	**0.99 (0.98–0.99)**	**0.042**	**0.98 (0.97–0.99)**	**0.029**

*DRE* digital rectal examination, *ISUP* International Society of Urological Pathology, *OR* odds ratio, *PI-RADS* Prostate Imaging Reporting and Data System, *PSA* prostate specific antigen, *ref.* reference, *95% CI* 95% confidence interval

^1^Step reflected as per increase of 1

## Discussion

Current guidelines recommend the combination of MRI-targeted and systematic bx as the diagnostic standard of care for men with suspected PCa on mpMRI. While there is growing evidence to suggest that omitting systematic bx may be safe in favor of MRI-targeted bx alone, little is known regarding the role of procedural experience in that context.

First, in this large sample of 576 men, increasing bx experience was associated with less benefit of systematic bx and lower risk of fusion failure on multivariable regression analysis. Second, a benefit of systematic bx was observed four times more often among men operated by residents in urology with very low procedural experience as compared to very experienced residents (18% versus 4%, *P* = 0.026). Consistently, fusion failures—evident in three fourths of the cases with the benefit of systematic bx (76% (48/63))—were nearly six times more common in residents with very low bx experience than in residents with very high bx experience (11% versus 2%, *P* = 0.015). Yet, the overall PCa detection rate was comparable across all experience levels in this study. These findings underscore the pivotal role of experience to minimize fusion failures and emphasize the value of systematic bx in less experienced urologists to ensure the detection of csPCa. In this regard, the most considerable value of the systematic bx seems to be the additional sampling of the prostate quadrant that has been deemed suspicious in the MRI. CsPCa (all ISUP 2) was detected by systematic bx outside these areas in less than 5% of biopsies. This indicates that MRI-negative csPCa might not be the most important reason for considering systematic bx. One possible future direction could be reducing systematically extracted bx cores by mainly focusing on additionally sampling the quadrant or prostate lobe entailing the suspicious MRI lesion. Interestingly, multi-regression analysis revealed that an increased number of targeted biopsies was associated with a higher likelihood of fusion failure. This might indicate that a higher number of extracted cores during the targeted bx might indicate uncertainty and improper fusion during the targeted bx. This stresses that in unexperienced urologists, a proper systematic bx of the suspicious quadrant or hemi prostate covering all regions might be of more value than a saturation during the targeted bx, which might be off-targeted due to improper software use or misinterpretation of the MRI image.

Probably the most challenging, experience-dependent aspect of MRI-targeted bx is the fusion of the patient’s mpMRI dataset with the live ultrasound image as it requires practice as well as an advanced understanding of the prostate’s anatomy. This may be particularly relevant when targeting smaller, less aggressive PCa [3]. This assumption is strengthened by the results of this current study. First, higher PSA levels were associated with lower risk of fusion failures on multivariable regression analyses. Second, most cases of csPCa identified on systematic, but not MRI-targeted biopsies were ISUP 2 PCa. This underscores that systematic biopsies may be most beneficial in men with small, less aggressive ISUP 2 PCa. In men with more aggressive PCa indicated by, e.g., a higher PSA value, the extent of the cancer infiltration of the prostate seems to be often underreported in the MRI. This, however, might lead to a lower likelihood of fusion failure in these patients as the detection rate of the targeted bx becomes less accuracy dependent.

Overall, 17% of all csPCa were diagnosed only with systematic bx leading to a benefit of systematic bx in 11% of all patients. This rate is comparable to data from other retrospective studies reporting a csPCa detection rate by systematic bx alone ranging from 6–16% [1, 4, 8, 17, 18, 30]. On the other hand, two prospective trials reported considerably lower rates of benefit from systematic bx following MRI-targeted bx (5 and 7% [23, 28] versus 11% in this current study). Yet, in both mentioned prospective trials as well as for the most recent GÖTEBORG-2 trial [11], all study related bx were performed by experienced urologists only and these rates of benefit for systematic biopsies were comparable to the results obtained by very experienced residents in this study (across all experience levels: 11% versus very experienced residents: 4%). These studies also reported that the added value of the MRI-targeted bx in detecting otherwise undetected csPCa (ISUP ≥ II) should be considered between 6–10% [5, 23, 30]. These findings are comparable with our results showing an overall benefit of targeted bx in 5% of patients. In fact, in very experienced residents the benefit of targeted bx (10%) was also higher than the benefit of systematic bx (4%) emphasizing the role of experience regarding MRI fusion biopsies of the prostate.

Previous studies suggested that systematic bx could be omitted in favor of targeted bx alone, particularly among patients with a (very) high risk for PCa (PI-RADS score = 5) [1, 4, 5]. Again, while this may hold true for a controlled study environment including experienced surgeons, the results of this study underscore the importance of overcoming learning curves inherent to MRI-targeted bx to ensure the quality of care for accurate risk stratification among less experienced surgeons. One previous study indicated that the learning curve for MRI-targeted bx may increase up to the 98th targeted bx [12]. Based on this retrospective analysis, performing more than 70 bx may be considered a critical, necessary threshold before considering an “MRI-target bx only” approach, despite convincing mpMRI findings.

Several limitations of this study are noteworthy. First, given its retrospective design the results of this study are prone to selection bias. Still, as no case was excluded during the investigated period, potential selection bias was minimized. Second, all prostate bx included in this analysis were performed transrectally. Thus, the results of this study might not be comparable to other studies investigating a transperineal approach. Third, the obtained experience thresholds were determined by visual binning and are thereby limited to the current sample and cannot be extrapolated to other collectives. Nevertheless, our analysis reflects a real-world scenario including data of 7 urologists in training with no prior bx experience. The analyses are further strengthened from a statistical/methodological perspective: the allocation of dynamic experience levels illustrates the growing procedural experience for every resident after every bx. From a clinical perspective, the detection rate of PCa by PI-RADS 4 or 5 lesions was 85% in this study and is not only comparable, but even higher than in most previous studies (results ranging from 40–80%) [3, 10, 12, 16, 18, 30]. These findings emphasize the overall high bx and mpMRI quality reported in this set of patients.

Taken together, the current study shows that bx experience potentially limits the generalizability of recent studies suggesting that systematic bx of the prostate can be omitted in favor of MRI-targeted approaches. The high risk of fusion failures among very inexperienced urologists warrants the addition of systematic bx following MRI-targeted bx to ensure the detection of csPCa in men with suspected PCa on mpMRI. Further prospective studies and the development of novel biopsy strategies are necessary to safely establish an accurate, minimally invasive and targeted only biopsy regime.

## Data Availability

Data are available for bona fide researchers who request it from the authors.
